# Analyzing Nursing Students’ Relation to Electronic Health and Technology as Individuals and Students and in Their Future Career (the eNursEd Study): Protocol for a Longitudinal Study

**DOI:** 10.2196/14643

**Published:** 2019-10-01

**Authors:** Peter Anderberg, Gunilla Björling, Louise Stjernberg, Doris Bohman

**Affiliations:** 1 Department of Health Blekinge Institute of Technology Karlskrona Sweden; 2 Department of Health Sciences The Swedish Red Cross University College Huddinge Sweden; 3 Department of Neurobiology, Care Sciences and Society Karolinska Institutet Stockholm Sweden; 4 Unit of Quality & Development Region of Blekinge Karlskrona Sweden

**Keywords:** internet, nursing, eHealth, nursing student, digitization

## Abstract

**Background:**

The nursing profession has undergone several changes in the past decades, and new challenges are to come in the future; patients are now cared for in their home, hospitals are more specialized, and primary care will have a key role. Health informatics is essential in all core competencies in nursing. From an educational perspective, it is of great importance that students are prepared for the new demands and needs of the patients. From a societal point of view, the society, health care included, is facing several challenges related to technological developments and digitization. Preparation for the next decade of nursing education and practice must be done, without the advantage of certainty. A training for not-yet-existing technologies where educators should not be limited by present practice paradigms is desirable. This study presents the design, method, and protocol for a study that investigates undergraduate nursing students’ internet use, knowledge about electronic health (eHealth), and attitudes to technology and how experiences of eHealth are handled during the education in a multicenter study.

**Objective:**

The primary aim of this research project is to describe the design of a longitudinal study and a qualitative substudy consisting of the following aspects that explore students’ knowledge about and relation to technology and eHealth: (1) what pre-existing knowledge and interest of this area the nursing students have and (2) how (and if) is it present in their education, (3) how do the students perceive this knowledge in their future career role, and (4) to what extent is the education capable of managing this knowledge?

**Methods:**

The study consists of two parts: a longitudinal study and a qualitative substudy. Students from the BSc in Nursing program from the Blekinge Institute of Technology, Karlskrona, Sweden, and from the Swedish Red Cross University College, Stockholm/Huddinge, Sweden, were included in this study.

**Results:**

The study is ongoing. Data analysis is currently underway, and the first results are expected to be published in 2019.

**Conclusions:**

This study presents the design of a longitudinal study and a qualitative substudy. The eHealth in Nursing Education eNursEd study will answer several important questions about nursing students’ attitudes toward and use of information and communications technology in their private life, their education, and their emerging profession. Knowledge from this study will be used to compare different nursing programs and students’ knowledge about and relation to technology and eHealth. Results will also be communicated back to nursing educators to improve the teaching of eHealth, health informatics, and technology.

**International Registered Report Identifier (IRRID):**

DERR1-10.2196/14643

## Introduction

### Background

This study presents the design, method, and protocol for a study that investigates undergraduate nursing students’ internet use, knowledge about electronic health (eHealth), and attitudes to technology and how their experiences of eHealth are handled during the education in a multicenter study. The nursing profession has undergone several changes in the past decades, and new challenges are to come in the future; patients are now cared for in their home, hospitals are more specialized, and primary care will have a key role. Health informatics and all forms of eHealth are essential parts of all core competencies in nursing. In this text, we will use the broad World Health Organization (WHO) definition of eHealth, “the use of information and communication technologies (ICTs) for health” [1].

Digitization can be described as a transformation, with far-reaching consequences in the lives of individuals as well as in the functioning of the society. Like almost all sectors of society, health care systems are increasingly characterized by digitalization and relying on technical aids and tools to solve basic assignments and needs. New technology can lead to great values in the form of safer and smoother care, increased quality of life, and socioeconomic efficiency improvements. The increased digitization and development in the eHealth area enables new ways of creating knowledge and conducting care, but information about people’s health is very sensitive, and incorrect handling of data can lead to serious consequences for the individual person. With the digitally available information, diseases can be prevented; medical quality and patient safety can be raised; and health care can be more efficient, coordinated, accessible, and transparent. To meet future demands, nursing students need to have knowledge about new technologies and eHealth. Therefore, there is a need for enhancing education in the field, although undergraduate students today are considered digital natives [2].

From a societal point of view, the society, health care included, is facing a number of challenges related to technological developments and digitization. Welfare technology is presented as 1 important way to meet this challenge: it can free resources, provide support to those who are in need, and decrease expenses. Furthermore, it is an important field for research, development, and innovation [3]. It is predicted that today’s dominating scenarios of human interaction and communication will, in the future, be complemented by an increase in the numbers of communicating machines. It is also expected that there will be 20 billion devices or things connected to the internet by 2020 [4]. Moreover, from an educational perspective, it is very important that students are prepared for the future demands and needs of the patients. Preparation for the next decade of nursing education and practice must be done without the advantage of certainty. A training for not-yet-existing technologies where educators should not be limited by present practice paradigms is desirable [5]. An important means to achieve the digitization goal is to increase digital literacy and competence in the society as a whole [6,7].

The Global Commission on Education of Health Professionals for the 21st century describes a lack of cooperation and discontinuous care [8], which poses demand on high-quality education in the field of technology in health care [9]. It is not only in high-income countries that the technological development is increasing tremendously but also in middle- and low-income countries where technological development is galloping, and the increased number of cellphone users and internet technologies has given new possibilities to reach areas that were not reachable before and people in developing countries. In addition, the technique is cheaper, which also enhances the number of users, including the older generation with new demands and possibilities [10].

In a report by WHO [11] about eHealth and how it can be beneficial for and support universal health coverage (UHC), it is stated that training of students as professionals in eHealth and electronic learning will contribute to reaching these goals for UHC. The report also points out that different areas within eHealth are rapidly increasing. Out of 109 responding countries, the vast majority (87%) answered that there is at least one mobile health (mHealth) program in their country. For low-income countries, the percentage was 80%, and for high-income countries, the percentage was 91%. There were several programs in the pipeline, but evaluations of these projects were few. In Sweden, the Government and the Swedish Association of Local Authorities and Regions have stated a vision for eHealth in Sweden [12]:

In 2025, Sweden will be best in the world at using the opportunities offered by digitization and eHealth to make it easier for people to achieve good and equal health and welfare and to develop and strengthen their own resources for increased independence and participation in the life of society.

Similar actions are taken globally; in Australia, the government of Queensland has undertaken a digital strategic vision for eHealth care including digital innovations [13], and in the European Union (EU), there are several initiatives for increasing and supporting digital transformation and digitization of health care [14]; for example, the European health telematics association, ETHEL, a platform for stakeholders that facilitates collaboration innovations and developments between health care and caregivers regarding digital health and care.

In the United States, there is an organization, the American Nursing Informatics Association (ANIA), whose goal is to improve care for patients and optimize management and communications for caregivers by delivering cost-effective, high-quality health care. In ANIA, nursing science, computer science, and information science are integrated [15]. Likewise, the Canadian Nurses Association (CNA) and the Canadian Nursing Informatics Association (CNIA) emphasize the importance of knowledge of nursing informatics within the nursing profession. CNA and CNIA point out that to reach a person-centered care-model, digitally connected health services environments are essential tools to empower the nurses in their caregiving [16]. In South Africa, with over 88 million mobile phone subscribers [17], the government has adopted an mHealth strategy to develop the health system and to transform health service delivery within the country to improve the efficiency of health care [18].

### Implications for the Nursing Profession

Health informatics is an important part in all core competencies in nursing [19]. In a survey from 21 different countries, different areas of health informatics were identified as key domains in health and nursing informatics. These were “data, information, knowledge,” “information exchange and information sharing,” “ethical and legal issues,” “systems life cycle management,” “management,” and “biostatistics and medical technology” [20]. The concept of health informatics is continuously developing [21], and the increasing digitalization of health care has identified the need for both general and specific digital competence and digital literacy in the nursing profession and education [22-24]. However, nursing informatics research on education, clinical practice, administration, and theory is scarce, with the theory being the least common [25]. Digitization has developed care practices, provides opportunities for the nurse to monitor patients at home, and facilitates control of patients, medications, etc [9,26,27]. WHO [28] defines digital health as general use of ICTs for health, including both internet- and mobile-based tools or apps aimed at treating and preventing diseases or promoting health and well-being. The complex process of integration of information and communications technology (ICT) into nursing practice impacts nurses’ communication and relationships in patient care as well as their working condition, professional identities, and development [29]. This has led to an increasing interest into various aspects of nurses’ ICT use, such as mobile phones in different care settings [30-33], social media [34,35], electronic records [36], Web-based guidelines [37,38], patient engagement technology [39], telehealth and telenursing [40], decision support [41], patient self-management [42], cybersecurity awareness [43], and multimedia Web-based simulation for competence development [44].

The importance of a structured approach to nurses’ use of eHealth has led nursing professional organizations in many countries to develop their own strategies in the area. In Sweden, the National Nursing Association (Swedish Nurses’ Association) has pointed out the importance of the necessary conditions and skills that are needed for added value both for patients and professionals from eHealth [45]. The strategy describes nurses’ responsibility for creating a relationship with patients and related parties based on a humanistic vision even when digital aids are used. It also highlights the importance of nurses participating to influence the development of the eHealth services nursing support.

### Implications for Nursing Education

It is necessary that nursing students are prepared to enter their professional role with knowledge and skills in eHealth. How nursing education is helping (or not) in this preparation and the strategies used to achieve this goal [46] is an important focus area. The reality that meets nursing students in their daily lives, in their education [47], and in their future career role is constantly changing in terms of the infrastructure they work within and the tools at their disposal. At the same time, the basic values and assignments are essentially the same [48]. This poses new ethical questions that need to be taken in to account [49]. In a study conducted by Montagni et al [50] about patterns of digital health use among university students in France, it was found that the students were confident that mobile apps and digital health interventions will replace physical consultations if they were launched by the government. Furthermore, the study shows that there is a potential for improving health and well-being among university students by using digital health interventions as the students are already using the internet for that purpose. These findings are confirmed by Covey and Potts [51] in a recent qualitative study with focus groups that aimed to understand undergraduate students’ experiences of both digital and broader health care to enable a better understanding of implications for national policy as well as for individual health care organizations. The study results also indicate that the apps need to be developed in collaboration for enhanced understanding and usefulness of the digital tools. These “digital native” students need to be convinced of the benefits of the tool before using it. In a Dutch study [52], however, this “internet-generation” nursing students were shown to not naturally have a positive view of technology-based health care provision. Although they could be seen as “digital natives,” they do not naturally display a positive view of technology-based health care provision, showing that adequate technology and telehealth education is still necessary.

Moreover, a study measuring students’ perceptions of the usefulness and ease of use of technology as a pedagogical tool in nursing education showed that they saw it as highly useful. In addition, use of social media in nursing education attracts interest as a learning platform [53-55] but is not seen as uncontroversial despite the potential benefits [56].

Early teaching of digital skills in undergraduate nursing students will enhance the development of digital professionalism among the students also in the health care environment [57]. These findings are essential for the implications of this study as they confirm that early digitization is important in the curriculum. This study presents the design, method, and protocol for a study that investigates undergraduate nursing students’ internet use, knowledge about eHealth, and attitudes to technology and how experiences of eHealth are handled during the education in a multicenter study.

### Objectives

eNursEd is a long-term research and development project aimed at describing, investigating, and analyzing nursing students’ relations to eHealth and technology as individuals and students and in their future career role. eNursEd is a collaborative project between the Blekinge Institute of Technology (Blekinge Tekniska Högskola, BTH) and the Swedish Red Cross University College (SRCUC) and is ongoing from 2017 onward.

The primary aim of the research project is to learn about the students’ knowledge about and relation to technology and eHealth. The project aims to investigate what pre-existing knowledge and interest of this area the nursing students have and how (and if) it is present in their education. In addition, the project aims to investigate how they perceive this knowledge in their future career role and, in particular, to what extent the education as such is capable of managing this knowledge.

## Methods

### Study Design and Setting

The study consists of 2 parts: a longitudinal study and a qualitative substudy.

The primary study has a longitudinal design, and data will be collected through a questionnaire distributed to all undergraduate nursing students at both universities. The data collection will be repeated every year; at the start of the semester, each fall, for as long as the project is active, but at least for the minimum duration of the project that is 7 years. The questionnaire will be distributed and gathered by research members who have no educational relationship with the students. Information to the students will emphasize the process of anonymity and the students’ right to withdraw from the study at any time. The questionnaire is distributed in a paper form to further the students’ feeling of anonymity, and the students are informed that they can choose to submit the questionnaire blank or choose to not submit it at all.

The questionnaire poses questions about general information technology (IT) usage, eHealth, and attitudes to technology and about how experiences of eHealth are handled during education. In addition to this quantitative part, a number of qualitative studies will be conducted where different types of questions will be highlighted from an in-depth perspective. To make global comparisons, the data collection will be broadened to include parts from low- and middle-income countries as well as from high-income countries to get a global perspective. Contacts with other partner universities in Europe, Africa, and the United States have already been taken and may expand the study in the future.

The participants are recruited from BTH, Karlskrona, Sweden, and from the SRCUC, Stockholm/Huddinge, Sweden.

There is a distinct focus on IT and innovation for sustainable growth. At BTH, the main focus is to contribute to more sustainable societal development through higher education, research, and innovation. BTH conducts education and research at a high international level, focusing on IT integrated with other subjects such as engineering, industrial economics, spatial planning, design, and health care. In the field of nursing, education is offered at the undergraduate level, that is, the bachelor level and an advanced level, including more than 500 students enrolled in the Bachelor of Nursing Science Program (3 years).

The SRCU, founded in 1867, was the first secular nursing education institution in Sweden. The SRCUC is owned by the nonprofit foundation for the Red Cross University College, an affiliated foundation to the Swedish Red Cross. It is, mainly, a state-funded self-governed university. It offers higher education in nursing at the undergraduate level, the bachelor level, and an advanced level. At present, there are over 750 students enrolled in the Bachelor of Nursing Science Program (3 years). In Table 1 the demographics of the two participating universities are presented.

**Table 1:** Demographics of the study setting.

University college	Blekinge Tekniska Högskola	Swedish Red Cross University College, Autumn 2017
Nursing students (n)	560	721
Weeks in clinical practice (n)	33	33
eHealth^a^ courses	Semester 3: eHealth^a^ 4.5 ECTS^b^; Semester 5: eHealth, Selectable Course 7.5 ECTS. All additional lectures within the Bachelor of Science (BSc) program	Included in preclinical courses in semester 3 and the semester within the BSc program
“City size” or number of inhabitants	Karlskrona: 66,796	Stockholm: 1,515,017 and Huddinge: 110,003

^a^eHealth: electronic health.

^b^ECTS is the European Community Course Credit Transfer System where 1.5 Credits corresponds to 1 week of fulltime studies.

### Inclusion Criteria

Undergraduate nursing students enrolled at the Bachelor of Nursing program at BTH and the SCRUC were eligible to participate in this study.

### Data Collection

#### Sociodemographic Data

This includes age, sex, education, and work experience before nursing studies.

#### Internet Use (Frequency and Type of Use)

Questions are drawn from a selected subset in 2 major investigations: the EU investigation into individuals’ use of ICT [58] and the World Internet Institute [59] common questions in collection of data on how people in different countries are using ICT and how this affects individuals, families, and society. This will allow for comparison with other populations. The possibility of creating scores from grouped variables will be evaluated and used in further analyses.

### Outcome Measures

#### Electronic Health Literacy Scale

The ability to seek out, find, evaluate and appraise, integrate, and apply electronic resources toward solving a health problem is called eHealth literacy [60,61]. eHeals is an 8-question instrument for self-reported eHealth literacy skills [60] and will be used in this study to evaluate the nursing students’ skills in using health technology. Stellefson et al [62] argue that the knowledge and skills necessary to conduct advanced eHealth searches are an important responsibility for the education community and that mere access to health resources does not ensure eHealth literacy. It can be a comparative advantage for nursing students to acquire the skills during their education for several benefits during their career. In a recent study [63], it was suggested that nurses with a high level of eHealth literacy had significantly positive overall health‐promoting behaviors and skills to help patients and families find up-to-date, reliable, and quality health information. Moreover, 2 previous studies have investigated nursing students’ eHealth literacy with the eHeals instrument in Jordan [64] and South Korea [65]. eHeals has been validated in other languages with a good result and will be translated into Swedish using backward-forward translation from the original English version. The construct validity of the Swedish version will be assessed in exploratory and confirmatory factor analyses, and internal consistency of the scale will be assessed using Cronbach alpha. A content validity index will also be created in the translation process.

#### Technophilia

For measuring the nursing students’ personal feelings and attitudes toward technology in general, a measurement of technophilia will be used [66]. Technophilia is generally seen as a strong enthusiasm for modern technology. In the study by Seebauer et al [67], it is defined as “an attitude towards ICT, representing a sub-aspect of technology-related values, just as ICT is a subcategory of modern technology.” Osiceanu [68] defines technophilia as an “attraction, enthusiasm of the human individual determined by the activities which involve the use of advanced technologies.” Martínez-Córcoles et al [69] suggest that merely enthusiasm and desire are not enough to define technophilia but also an acquired need for (dependency), and joy of having the latest products and versions (technoreputation). According to Seebauer et al [67], openness toward technology and innovation influences personal dedication to certain technological artifacts and services, whereas feelings of low enthusiasm may work in the opposite direction.

#### Technology and Electronic Health in Nursing Education

This section comprises 2 components, each with 3 questions. The first component concerns how nursing students perceive the scope and content of eHealth in their current education. The second component deals with their perception of the importance and necessity of the knowledge of eHealth and their coming profession as nurses.

### Statistical Analyses

Baseline characteristics will be tabulated using standard descriptive statistics, and bivariate correlations between variables will be analyzed with parametric and nonparametric measures. Additional data analysis (*t* test and analysis of variance [ANOVA]) will be used to investigate differences in eHealth and health literacy according to sociodemographic characteristics of the participants. The association between dependent variables and independent variables (health literacy, techPH, etc) will also be analyzed in various regression models before and after adjusting for background variables. To analyze changes over time, we will use instruments for longitudinal data analysis, for example, repeated measurements ANOVA.

In the validation process of the Swedish translation of eHeals, content validity, construct validity, and internal consistency will be assessed using exploratory and confirmatory factor analyses and internal consistency reliability analysis using Statistical Package for the Social Sciences (SPSS) version 25 (IBM Corp) and exploratory and confirmatory factor analyses with SPSS AMOS.

### Qualitative Inquiry Substudy

The substudy has a qualitative design, and a subset of participating students at each university will be asked to participate in a substudy.

A separate study protocol and analysis plan will be developed before starting the qualitative substudy. The qualitative enquiry substudy will provide the perceptions and experiences from the individual participant’s point of view to further develop knowledge based on the results and findings of the quantitative study.

An interview guide will be used consisting of a general question “Can you please tell me about your perception or experience of eHealth?” followed by more specific questions on private versus educational experiences of eHealth and probe questions such as “Tell me more…Can you explain further...Elaborate on that...”

### Ethics

The questionnaire does not collect sensitive data that can be linked to any individual and is anonymous. The students can choose not to participate in the study. As students are a vulnerable population, the anonymous questionnaires were distributed and gathered by research members who had no educational relationship with the students. Information was emphasized on the process of anonymity and the students’ right to withdraw from the study at any time.

Regarding the qualitative substudy, students will be invited by means of an information letter handed out by the research team. Those who accept to participate will receive verbal and written information about the study and information on their right to withdraw at any time. To ensure compliance with the Data Protection Act, data will be coded to protect and ensure the participants’ anonymity and will be stored securely.

For the qualitative substudy, ethical approval will be obtained from the regional committees of ethics in Stockholm and south Sweden, depending on the issues that will be handled in this part of the study. Written informed consent will be obtained from the participants in the substudy. Confidentiality will be ensured for all participants in the project.

The results will be published in scientific journals but will also be made available in such a way that they can be used for feedback to the educational development work at the respective academic institutions.

The database used for the unidentified data is located physically at BTH and is used for several other clinical studies, including the Swedish National Study of Aging and Care, following all relevant protocols for data security and integrity.

### Dissemination

Data will be presented on the group level only, ensuring that individual participants cannot be identified. The findings will be published in national and international peer-reviewed scientific journals and presented at relevant scientific conferences. The deidentified data will be posted in an open access data repository in accordance with the requirements of the scientific journal. Authorship will be determined in accordance with the International Committee of Medical Journal Editors guidelines.

## Results

The study is ongoing. Data analysis is currently underway, and the first results are expected to be published in 2019.

## Discussion

This study describes a project aimed at investigating nursing students’ knowledge and attitudes to digitization and eHealth. The eNursEd study will answer several important questions about nursing students’ attitudes toward and use of ICT in their private life, their education, and their emerging profession. Some questions in this study have been addressed previously and are in focus in other ongoing research projects. A special feature of this study is that several different factors are studied at the same time, with a large number of participants and the long time span over which they will be followed. This allows comparison and measurement of progress throughout the entire education. It will also give answers about how the use of internet and technology develops in separate groups over time.

Knowledge from this study will be used to compare different nursing education programs and students’ knowledge about and relation to technology and eHealth. Results will also be communicated back to nursing education to improve the teaching of eHealth, health informatics, and technology in a nursing curriculum.

## Abbreviations

ANIA: American Nursing Informatics Association
BTH: Blekinge Tekniska Högskola
CNA: Canadian Nurses Association
CNIA: Canadian Nursing Informatics Association
eHealth: electronic health
EU: European Union
ICT: information and communication technology
IT: information technology
mHealth: mobile health
SRCUC: Swedish Red Cross University College
UHC: universal health coverage
WHO: World Health Organization
